# Antibiotic distribution channels in Thailand: results of key-informant interviews, reviews of drug regulations and database searches

**DOI:** 10.2471/BLT.17.199679

**Published:** 2018-01-02

**Authors:** Angkana Sommanustweechai, Sunicha Chanvatik, Varavoot Sermsinsiri, Somsajee Sivilaikul, Walaiporn Patcharanarumol, Shunmay Yeung, Viroj Tangcharoensathien

**Affiliations:** a London School of Hygiene & Tropical Medicine, Keppel Street, London WC1E 7HT, England.; b International Health Policy Programme, Ministry of Public Health, Nonthaburi 110000, Thailand.; c Food and Drug Administration, Ministry of Public Health, Nonthaburi, Thailand.; d Department of Livestock Development, Ministry of Agriculture and Cooperatives, Phathumtani, Thailand.

## Abstract

**Objective:**

To analyse how antibiotics are imported, manufactured, distributed and regulated in Thailand.

**Methods:**

We gathered information, on antibiotic distribution in Thailand, in in-depth interviews – with 43 key informants from farms, health facilities, pharmaceutical and animal feed industries, private pharmacies and regulators– and in database and literature searches.

**Findings:**

In 2016–2017, licensed antibiotic distribution in Thailand involves over 700 importers and about 24 000 distributors – e.g. retail pharmacies and wholesalers. Thailand imports antibiotics and active pharmaceutical ingredients. There is no system for monitoring the distribution of active ingredients, some of which are used directly on farms, without being processed. Most antibiotics can be bought from pharmacies, for home or farm use, without a prescription. Although the 1987 Drug Act classified most antibiotics as “dangerous drugs”, it only classified a few of them as prescription-only medicines and placed no restrictions on the quantities of antibiotics that could be sold to any individual. Pharmacists working in pharmacies are covered by some of the Act’s regulations, but the quality of their dispensing and prescribing appears to be largely reliant on their competences.

**Conclusion:**

In Thailand, most antibiotics are easily and widely available from retail pharmacies, without a prescription. If the inappropriate use of active pharmaceutical ingredients and antibiotics is to be reduced, we need to reclassify and restrict access to certain antibiotics and to develop systems to audit the dispensing of antibiotics in the retail sector and track the movements of active ingredients.

## Résumé

**Objectif:**

Analyser l'importation, la fabrication, la distribution et la réglementation des antibiotiques en Thaïlande.

**Méthodes:**

Nous avons rassemblé des informations sur la distribution des antibiotiques en Thaïlande à partir d'entretiens approfondis avec 43 informateurs clés –provenant d'exploitations agricoles, d'établissements de soins, du secteur pharmaceutique et de la production d'aliments pour animaux, de pharmacies et d'organismes de réglementation privés– et de recherches dans des publications et des bases de données.

**Résultats:**

En 2016-2017, la distribution d'antibiotiques autorisés en Thaïlande a fait intervenir plus de 700 importateurs et environ 24 000 distributeurs, comme les pharmacies d'officine et les grossistes. La Thaïlande importe des antibiotiques et des principes actifs pharmaceutiques. Elle n'a pas de système de contrôle de la distribution des principes actifs, dont certains sont utilisés directement dans les exploitations agricoles, sans traitement préalable. La plupart des antibiotiques sont en vente dans les pharmacies, pour usage domestique ou agricole, sans ordonnance. Bien que la Loi sur les médicaments de 1987 ait classé la plupart des antibiotiques comme «médicaments dangereux», elle n'en a classé qu'une petite partie en tant que médicaments soumis à ordonnance et n'impose aucune restriction sur la quantité d'antibiotiques qui peut être vendue à une personne. Certaines dispositions de cette Loi s'appliquent aux pharmaciens qui travaillent dans des pharmacies, mais il apparaît que la qualité de leur délivrance et de leur prescription dépend fortement de leurs compétences.

**Conclusion:**

En Thaïlande, la plupart des antibiotiques sont très facilement accessibles dans les pharmacies d'officine, sans ordonnance. Si l'on veut réduire l'usage inapproprié des principes actifs pharmaceutiques et des antibiotiques, il faudra reclasser certains antibiotiques et en limiter l'accès, mettre au point des systèmes pour contrôler la délivrance d'antibiotiques dans les officines et contrôler les mouvements des principes actifs.

## Resumen

**Objetivo:**

Analizar cómo se importan, fabrican, distribuyen y regulan los antibióticos en Tailandia.

**Métodos:**

Recopilamos información sobre la distribución de antibióticos en Tailandia, en entrevistas en profundidad, con 43 informadores clave de granjas, centros de salud, la industria farmacéutica y alimentación animal, farmacias privadas y reguladores, y en búsquedas de bases de datos y bibliografía.

**Resultados:**

En 2016-2017, en la distribución autorizada de antibióticos en Tailandia participan más de 700 importadores y alrededor de 24 000 distribuidores, p.e. farmacias minoristas y mayoristas. Tailandia importa antibióticos y sustancias farmacéuticas activas. No existe un sistema para controlar la distribución de los ingredientes activos, algunos de los cuales se usan directamente en las granjas, sin ser procesados. La mayoría de los antibióticos se pueden comprar en farmacias, para uso doméstico o agrícola, sin receta médica. Aunque la Ley de Medicamentos de 1987 clasificó la mayoría de los antibióticos como “drogas peligrosas”, solo clasificó algunos de ellos como medicamentos de venta con receta y no restringió las cantidades de antibióticos que podrían venderse a cada persona. Los farmacéuticos que trabajan en farmacias están cubiertos por algunas de las normas de la Ley, pero la calidad de su dispensación y prescripción parece depender en gran medida de sus competencias.

**Conclusión:**

En Tailandia, la mayoría de los antibióticos están disponibles de forma fácil y general en farmacias minoristas, sin receta médica. Para reducir el uso inapropiado de sustancias farmacéuticas activas y antibióticos, debemos volver a clasificar y restringir el acceso a ciertos antibióticos y desarrollar sistemas para auditar la dispensación de antibióticos en el sector minorista y llevar a cabo un seguimiento de los movimientos de los ingredientes activos.

## ملخص

**الغرض:**

تحليل كيفية استيراد المضادات الحيوية وتصنيعها وتوزيعها وتنظيمها في تايلند.

**الطريقة:**

قمنا بجمع معلومات حول توزيع المضادات الحيوية في تايلند في مقابلات متعمقة مع 43 من المبلغين الرئيسين من المزارع والمنشآت الصحية وصناعات المنتجات الدوائية وعلف الحيوانات والصيدليات الخاصة ومنظمين، وكذلك في قاعدة البيانات وأبحاث المؤلفات.

**النتائج:**

في عام 2016-2017 تضمن التوزيع المرخص للمضادات الحيوية في تايلند أكثر من 700 مستورد وحوالي 24 ألف موزع مثل الصيدليات التي تبيع بالتجزئة للجمهور وبائعي الجملة. تستورد تايلند المضادات الحيوية والمكونات الدوائية النشطة دون وجود نظام لمراقبة توزيع المكونات النشطة، والتي يُستخدم بعضها مباشرًة في المزارع دون خضوعها للمعالجة. ومن الممكن شراء معظم المضادات الحيوية من الصيدليات لاستخدامها في المنازل أو المزارع دون وصفة طبية. وعلى الرغم من أن قانون العقاقير لعام 1987 جاء ليصنف أغلب المضادات الحيوية "كعقاقير خطيرة"، فقد صنف فقط القليل منها كعقاقير لا تُصرف إلا بأمر الطبيب، دون أن يفرض أي قيود على كميات المضادات الحيوية المصرّح ببيعها لأي فرد. وهناك بعضٌ من اللوائح التنفيذية للقانون التي تغطي الصيادلة العاملين في الصيدليات إلا أنه يبدو أن جودة وصفهم للأدوية وصرفها تتوقف بشكل كبير على مستوى كفاءتهم.

**الاستنتاج:**

تتوفر معظم المضادات الحيوية في تايلند بسهولة وعلى نطاق واسع لدى الصيدليات التي تبيع للجمهور دون وصفة طبية. وإذا كان من المعتزم الحد من الاستخدام غير الأمثل للمكونات الدوائية النشطة والمضادات الحيوية، فسوف يلزمنا إعادة تصنيف مضادات حيوية معينة وفرض قيود على صرفها، فضلاً عن وضع أنظمة لمراجعة صرف المضادات الحيوية في قطاع البيع بالتجزئة، وتتبع أوجه استخدام المكونات النشطة.

## 摘要

**目的:**

分析泰国的抗生素是如何进口、加工、销售和监管的。

**方法:**

我们收集了泰国的抗生素销售信息，深入采访了来自农场、医疗机构、制药和动物饲料行业、私人药店和监管机构的 43 名关键知情人，并进行了数据库和文献检索。

**结果:**

2016-2017 年，泰国获得许可的抗生素销售涉及 700 多家进口和 24000 家分销商——例如零售药店和批发商。泰国进口抗生素和活性药物成分。目前还没有用于监测活性成分的分配的系统，其中一些未经过处理而直接在农场使用。大多数抗生素可以从药店购买，供家庭或农场使用，无需处方。尽管 1987 年的《药品法》将大多数抗生素列为“危险药物”，但它只将其中少量的抗生素列为处方类药物，并未对可以出售给任何个人的抗生素的数量加以限制。药店工作的药剂师受该法案中的某些条例的监管，但是其配药和开药的质量似乎在很大程度上依赖于他们的能力。

**结论:**

在泰国，大多数抗生素很容易从零售药店买到，无需处方。如要减少对活性药物成分和抗生素的不当使用，我们需要对某些抗生素进行重新分类和限制销售，并开发相应系统审核零售部门的抗生素分配和追踪活性成分的流通。

## Резюме

**Цель:**

Выяснить, каким образом происходит импорт, производство, распространение и контроль антибиотиков в Таиланде.

**Методы:**

Мы собрали информацию о распространении антибиотиков в Таиланде с помощью углубленных интервью с 43 ключевыми информантами — представителями фермерских хозяйств, медицинских учреждений, фармацевтической и комбикормовой промышленности, частных аптек и регулирующих органов, а также путем поисках в базах данных и научной литературе.

**Результаты:**

В 2016–2017 гг. сеть лицензированного распространения антибиотиков в Таиланде включала более 700 импортеров и около 24 000 дистрибьюторов, таких как розничные аптеки и организации оптовой торговли. Таиланд импортирует антибиотики и активные фармацевтические ингредиенты. При этом в Таиланде отсутствует система мониторинга распространения активных ингредиентов, некоторые из которых в исходном виде используются непосредственно на фермах. Большинство антибиотиков можно купить для домашнего или фермерского использования в аптеках без рецепта. Хотя в Законе о продаже рецептурных лекарственных средств (Prescription Drug Marketing Act) от 1987 года большинство антибиотиков классифицированы как сильнодействующие лекарственные средства, в то же время в этом документе лишь некоторые из них классифицированы как лекарственные средства, отпускаемые по рецепту, и отсутствуют ограничения по количеству антибиотиков, в котором они могут быть отпущены одному лицу. Некоторые положения закона распространяются на фармацевтов, работающих в аптеках, но качество их назначения и отпуска лекарственных средств, по-видимому, в значительной степени зависит от их компетенции.

**Вывод:**

В Таиланде большинство антибиотиков легко и широко доступны в розничных аптеках, где их можно приобрести без рецепта. Чтобы сократить ненадлежащее использование активных фармацевтических ингредиентов и антибиотиков, необходимо повторно их классифицировать, ограничить доступ к определенным антибиотикам и разработать системы контроля отпуска антибиотиков в розничном секторе и отслеживания движения активных ингредиентов.

## Introduction

To address antimicrobial resistance, antibiotics should be used appropriately in human medicine. Patients should receive antibiotics “appropriate to their clinical needs, in doses that meet their own individual requirements, for an adequate period of time”.^4^ Similar rules apply to the prudent use of antibiotics by all of the relevant stakeholders involved in veterinary medicine.^5^

The inappropriate use of antibiotics may involve the use of antibiotics for a health problem for which antibiotics are not indicated or the rational use of antibiotics either in doses that are inadequate or in the correct doses, but for an inadequate duration. As exposure of susceptible bacteria to low doses of antibiotics can lead to the selection of resistance,^1^ there is a strong association between antimicrobial resistance and inappropriate use of antibiotics at both individual and population levels.^2,3^

In most developing countries, many antibiotics can be easily bought without prescription and self-medication with antibiotics, mostly bought from drugstores or pharmacies or left over from previous treatments, is common.^6,7^ Such self-medication is also found in some high-income countries.^8^

A major aim of the pharmaceutical market is to respond to increased demand. As the number of retail pharmacies and other outlets for the distribution of antibiotics increases, antibiotics become more widely and easily available. Health professionals may also be persuaded to over-prescribe antibiotics by financial incentives.^9^

In low- and middle-income countries most drug regulation is focussed on the quality of drugs and the process of licensing; relatively little attention is given to distribution, price and other aspects of market control. Furthermore, in such countries, the enforcement of the drug regulations that do exist is often poor and the sale of substandard over-the-counter antibiotics and weak pharmaco-vigilance are often common.^10–12^

One of the main aims of the Global Action Plan on Antimicrobial Resistance, which was adopted by the World Health Assembly in 2015, was to optimize the use of antibiotics in human and veterinary medicine.^13^ A key goal of Thailand’s subsequent National Action Plan on Antimicrobial Resistance, which was developed and endorsed by the Thai Cabinet in 2016, was to reduce antibiotic consumption, by 20% in human medicine and by 30% in veterinary medicine by 2021.

In 2009, the value of the antibiotics imported into Thailand or manufactured in the country was about 315 million United States dollars and this value represented about 10% of the total value of the medicines consumed in the country.^15^ There appears to be widespread and often unregulated use of antibiotics, not only for human and pet health, but also for the treatment of livestock both on farms and in household settings.

In 2016, we decided to investigate Thailand’s importation, manufacture, distribution and regulation of antibiotics. In interviews with key informants, we investigated the multiple channels for the distribution of antibiotics, from import and manufacture to retail sale, and the various issues that probably contribute to the inappropriate use of antibiotics.

## Methods

We investigated antibiotic distribution and regulation in Thailand using a combination of key-informant interviews, a review of the relevant drug regulations and database searches.

### Interviews

Between the July and November of 2016, we conducted in-depth interviews, lasting a mean of 90 minutes, with 43 key informants. Each interviewee had been selected using a purposive sampling technique in which relevant associations, i.e. Thailand’s Animal Health Products, Animal Feed Mill, Community Pharmacy and Pharmaceutical Manufacturers Associations, were asked to propose lists of their members who could provide information about antibiotic distribution. Each potential informant identified was asked if they were able and willing to participate in the study and, if so, they were asked to give their written informed consent. Our initial aim was to recruit at least three consenting informants from each of six main stakeholder groups, i.e. animal feed industries, farms, government authorities in the fields of human and animal health, health facilities, pharmaceutical industries and pharmacies. However, using the snowball technique, more key informants were interviewed until our data became saturated and no new information emerged (Table 1). To ensure consistency, the same individual (AS) interviewed each key informant.

**Table 1:** Types, ages and years in relevant work of the 43 key informants, Thailand, 2016

Type	No. of informants	Ages (years)	Relevant work experience (years)**^a^**	
			Mean	Range
Regulator	13	35–59	15.9	0.5–32.0
Representative of pharmaceutical company^b^	14	35–65	17.1	3.0–40.0
Representative of animal feed company^c^	5	30–61	18.5	3.5–37.0
Health professional from human or animal health facility	4	35–54	14.3	1.0–31.0
Wholesaler or owner of retail drug store	4	36–70	25.5	11.0–42.5
Farmer	3	37–52	16.6	13.0–19.0
Total	43	30–70	17.2	0.5–42.5

^a^ Recorded after rounding to the nearest half year.

^b^ Involved in the import, manufacturer and/or distribution of antibiotics.

^c^ Running a feed mill or feed store.

All of the interviews were conducted face-to-face, in Thai. They were semi-structured, but based on open-ended questions. The informants were asked about the processes of antibiotic import, manufacturing, distribution, dispensing, prescription and use. For example, they were asked about the sources of active pharmaceutical ingredients used in the manufacture of finished products and about their sale patterns. All of the interviewees were asked about the licensing process and requirements for each distributor, the registration of medicines and the factors that might contribute to the excessive and inappropriate use of antibiotics. The informants representing the farming industry or health facilities were asked about their sources of antibiotics and the processes they followed to purchase such drugs or active pharmaceutical ingredients. The data recorded in each interview were kept confidential.

### Database searches

We estimated the numbers of licensees involved in antibiotic distribution in the Thai market and in the regulation of such distribution by analysing the relevant databases held by the Thai Food and Drug Administration^32^ and the Thai Department of Livestock Development.^14^

### Drug regulations

We reviewed all of the regulations promulgated by both of the Acts that, in 2016, regulated the use of antibiotics and medicated feed through inspection, licensing and marketing: the 1987 Drug Act^30^ and the 2015 Animal Feed Quality Control Act.^31^ The 1987 Drug Act, enforced by the Food and Drug Administration of the Thai Ministry of Public Health, regulates the finished products used in human and veterinary medicine and active pharmaceutical ingredients. The 2015 Animal Feed Quality Control Act is enforced by the Department of Livestock Development of the Thai Ministry of Agriculture and Cooperatives. 

### Data analysis

The data obtained from the key-informant interviews and document reviews were summarized to provide an overview of the distribution of antibiotics and identify weaknesses that could contribute to the inappropriate use of antibiotics. To assess the accuracy of the interview data, we used triangulation across the 43 interviewees. If information from one interviewee differed substantially from, and contradicted, the corresponding information from another interviewee, both pieces of information were ignored. Thailand’s antibiotic distribution channels were summarized as a system flowchart. The provincial numbers of licensed private pharmacies per 100 000 population were mapped using ArcGIS software (Esri, Redlands, United States of America).

### Ethics

The study protocol was approved by the Research Ethics Committee at the Thai Ministry of Public Health’s Institute for Development of Human Research. Interviewees gave their written informed consent. Strict confidentiality was observed and interviewees could opt out from the interviews at any time.

## Results

**Fig. 1:**
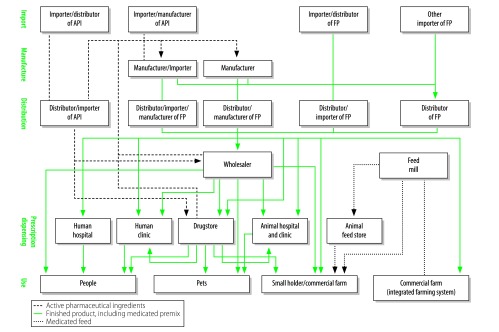
Antibiotic distribution channels, Thailand, 2016

API: active pharmaceutical ingredients; FP: finished product.

Note: An integrated farming system covers all aspects of the commercial production of livestock, including breeding, feeding, processing and marketing.

We created a flowchart, based on data from the key-informant interviews and reviews of the 1987 Drug Act and the 2015 Animal Feed Quality Control Act, to summarize the antibiotic distribution channels (Fig. 1). It illustrates the complexity of the distribution, of active pharmaceutical ingredients, finished products and medicated feed, from the importers and local manufacturers to final consumption by humans, livestock or pets**.**

### Import, manufacture and distribution

Thailand imported active pharmaceutical ingredients, for local manufacturing into finished products. It also imported medicated premix for the manufacture of medicated feed by feed mills. Active pharmaceutical ingredients were imported either by manufacturers or by licensed importers that then sold the ingredients to manufacturers. Most of the manufacturers either purchased active pharmaceutical ingredients from licensed importers or imported such ingredients themselves – rather than buying them, at a greater cost, from drugstores. The antibiotics produced by the manufacturers were sold to distributors, retail outlets and/or wholesalers. The imported finished products were distributed, by importers who were licensed to distribute or by distributors, to drugstores, farms, feed mills, health facilities, veterinary facilities and/or wholesalers. Our data indicated that the import and manufacture of human medicines were very similar to those of veterinary medicines, because the Thai Food and Drug Administration regulated all of these processes.

Several interviewees, representing regulators, retailers and wholesalers, described the illegal distribution of both finished products and certain active pharmaceutical ingredients. The 1987 Drug Act stipulates that all active pharmaceutical ingredients must be used by manufacturers to produce finished products. However, a few informants reported how drug inspectors had confiscated active pharmaceutical ingredients that were being used directly on livestock in farms. The interviewees that represented the farming industry reported how the high cost of buying medicated feed had persuaded some farmers to mix active pharmaceutical ingredients into their animal feed. The farmers who produced their own medicated feed did not have quality control and, in the interviewees’ opinion, the feed they produced was unlikely to have an even distribution of active pharmaceutical ingredients. Although the 2015 Animal Feed Quality Control Act prohibited such direct use of active pharmaceutical ingredients in animal feed, inadequate inspection allowed farmers to purchase such ingredients from drugstores or wholesalers.

According to the various ministerial notifications and regulations promulgated by the 1987 Drug Act, most antibiotics are classified as “dangerous drugs” that can only be dispensed by licensed pharmacists in pharmacies, but can be obtained, legally, without a prescription. Only a few antibiotics, e.g. betalactamase inhibitor, carbapenems and fosfomycin, are classified as special-control drugs because of the high prevalence of resistance to them. Such drugs cannot be obtained, legally, without a prescription and are reserved for hospital use.

According to our interviews with key informants representing the country’s health providers, every private and public clinic and hospital had a pharmacy section in which antibiotics were dispensed to inpatients and outpatients according to the prescriptions of doctors. Although most of these prescriptions were not required by law, the routine issuing of prescriptions, even for drugs that were not, legally, prescription-only, had become the tradition of most health facilities. Antibiotics were also dispensed directly to consumers and pet owners by licensed pharmacists in wholesalers or drugstores.

Informants representing animal feed companies reported how feed mills mostly purchased medicated premix, from importers, manufacturers or distributors, to produce medicated feed that was then sold to farms either directly or via feed stores. According to the key informants from the farming industry, most of the antibiotics that farmers used were given to livestock in medicated feed, either for treatment or for prophylaxis during periods of increased vulnerability, e.g. when livestock were transferred to new environments.

**Fig. 2:**
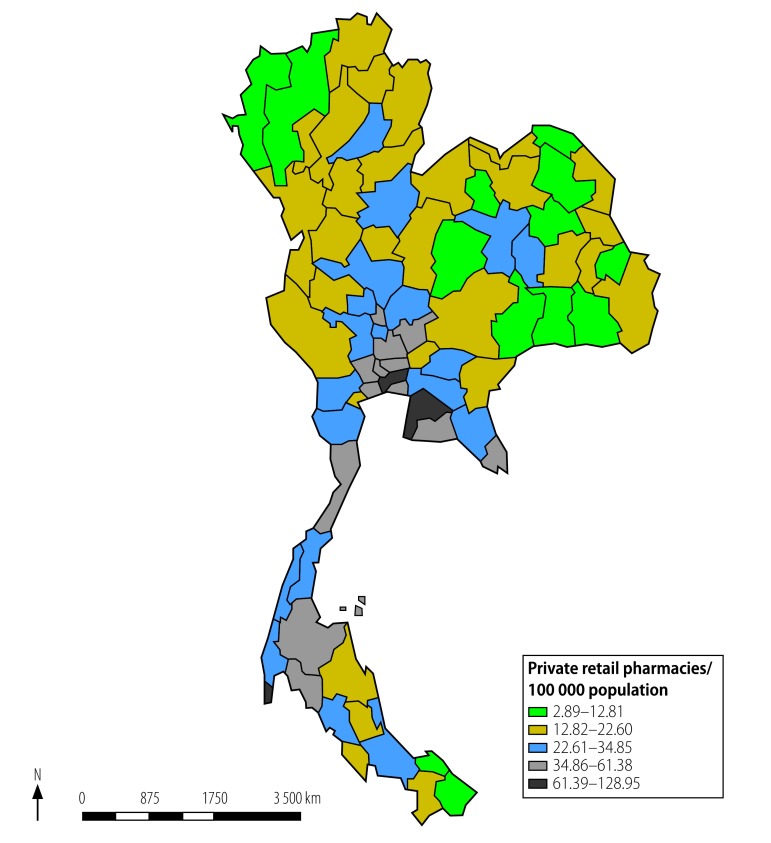
Provincial numbers of private licensed retail pharmacies per 100 000 population, Thailand, 2016

Source: Based on data from the Thai Food and Drug Administration’s records for 2017.^32^

The large number of licensed individuals involved in the antibiotic supply chains can be categorized according to the type of license granted to them under the 1987 Drug Act or 2015 Animal Feed Quality Control Act. According to the licenses issued in 2016–2017, these chains involved 793 drug importers, 187 drug manufacturers, 323 animal feed importers, 299 animal feed mills, 27 165 feed stores and about 24 000 other individuals who were distributors, wholesalers or retail pharmacies (Table 2). Of the 793 importers involved in antibiotic distribution, 675 (85%) were located in Bangkok, the capital city where the main air and sea ports are located.^32^ From Bangkok, many medicines, including antibiotics, are distributed throughout the country by importers, manufacturers and wholesalers, with sales driven, as usual, by market forces. In 2016, the provinces of Bangkok, Chonburi and Phuket had more than 61 licensed private pharmacies per 100 000 population (Fig. 2).

**Table 2:** The types and numbers of individuals involved in the distribution of antibiotics and other medicines, Thailand, 2016–2017

Type	License held	No. of individuals
**Licensed providers **		
Medicine importers	Pharmaceutical import	793^a^
Medicine manufacturers	Pharmaceutical manufacture	187^a^
Medicine distributors	Pharmaceutical sales	NA^a^
Medicine wholesalers	Pharmaceutical sales	NA^a^
Retail drug stores or pharmacies		
Selling all medicines	Pharmaceutical sales	NA^a^
Selling only ready-packed medicines	Pharmaceutical sales – ready-packed medicines only	3164^a^
Selling only ready-packed medicines for animals	Pharmaceutical sales – ready-packed medicines for animals only	763^a^
Human health facilities	Health facility	11 560^b^
Importers of animal feed	Animal feed import	323^c^
Animal feed mills	Animal feed manufacture	299^c^
Animal feed stores	Animal feed sales	27 165^c^
Animal health facilities	Animal health facility	2058^d^
**Unlicensed individuals**		
Households involved in the rearing of livestock	None	3 102 530^e^

NA: not available.

^a^ In 2017, according to the Thai Food and Drug Administration’s records, there were 19 934 individuals holding full pharmaceutical sales licenses in Thailand.^32^

^b^ Data from the Thai Ministry of Public Health’s records for 2016.^33^

^c^ Data from the Thai Department of Livestock Development’s records for 2016.^35^

^d^ Data from the Thai Department of Livestock Development’s records for 2016.^36^

^e^ The estimated number of households involved in the rearing of livestock.^14^

Our database searches revealed how, in 2015, about 3.1 million Thai households raised chickens (*n* = 2.4 million), ducks (*n* = 0.4 million), buffalo (*n* = 0.2 million) and/or pigs (*n* = 0.2 million).^14^

### Market authorization and licensing

Overall, 5371 antibiotics were registered in the Thai Food and Drug Administration’s database for 2016.^34^ Of these, 3371 (63%) were registered for human use and the rest for use on livestock and pets, some as medicated premix. The database records did not distinguish between imported antibiotics and those produced in Thailand.

The importation of any drugs must be registered and pre-approved by the Thai Food and Drug Administration. By law, active pharmaceutical ingredients must only be sold by licensed importers and manufacturers. At customs, the licensed importers of active pharmaceutical ingredients are required to notify the Thai Food and Drug Administration before gaining approval for imports.

The 1987 Drug Act regulates pharmacists working in pharmacies, on aspects such as working hours and the dispensing of special-control drugs. However, most of the dispensing of antibiotics classified as dangerous drugs is not legally regulated and the quality of dispensing is largely reliant on the competences of the doctors, pharmacists and veterinarians involved. Historically, there have been no legal requirements for the keeping of records on the types and quantities of antibiotics dispensed within the retail sector. At the time of our study, prescriptions were routinely issued in hospitals, but no prescription audits were required.

## Discussion

**Fig. 3:**
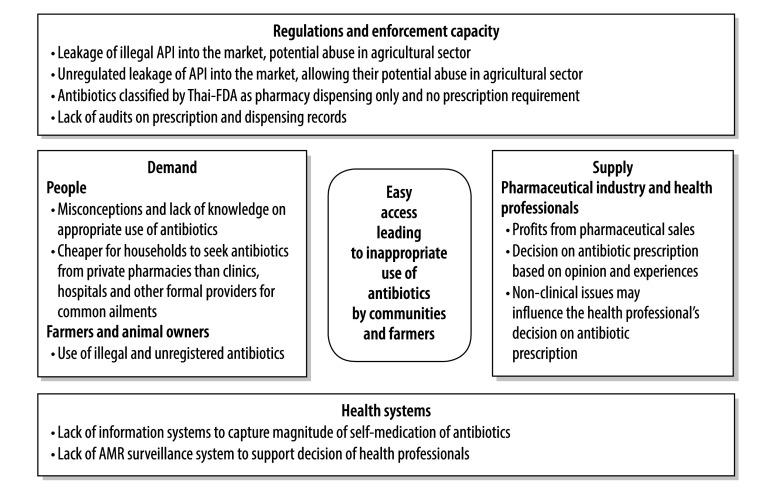
Factors potentially contributing to the excessive and/or inappropriate use of antibiotics, Thailand, 2016

AMR: antimicrobial resistance; API: active pharmaceutical ingredients; FDA: Food and Drug Administration.

Our study was triggered by the Global Action Plan on Antimicrobial Resistance. In this study, we identified a few key challenges, on both the demand and supply sides of the market as well as in health facilities and the regulatory environment, that perhaps made access to antibiotics too easy (Fig. 3).

### Demand by patients and farmers

Self-medication with antibiotics obtained without a prescription is a common practice in most developing countries.^16^ Although such self-medication may appear to be a relatively cheap option for the sick and their household carers, the societal cost of such treatment, often associated with inappropriate drugs or appropriate drugs in inadequate or suboptimal doses, and with a lack of counselling by the drug provider, can be relatively high. In China and Viet Nam, inadequate knowledge and lack of awareness of antimicrobial resistance, in both patients and providers, were recognized as important factors contributing to the irrational use of antibiotics.^17^ Inadequate regulation of drug distribution and sales may result in easy access, especially when, as is often the case in Thailand, prescriptions are not required. In turn, easy access may boost the inappropriate use of drugs by households.^18,19^

### Supply problems

Any economic incentives offered by pharmaceutical companies to boost their market share may contribute to the excessive provision of antibiotics.^17^ Some pharmaceutical companies support clinicians by sponsoring continuing professional education, financing international travel for conferences and leisure or offering generous speaking fees.^20–22^ In an attempt to break the link between such incentives and the preferential dispensing of drugs produced by the company providing the incentives, Denmark has decoupled the prescribing and dispensing of medicines by veterinarians.^23^ Almost all medicines used in the livestock sector in Denmark are now sold directly to the farmers by pharmacies.^23^

In much of Asia, the quality of the pharmaceutical services provided by retail pharmacies is often poor. The staff in such pharmacies may offer no counselling or history taking and may recommend inappropriate presumptive treatments, e.g. antibiotics for the treatment of the symptoms of a common cold or influenza, or appropriate drugs in suboptimal doses.^24^ Suboptimal doses may be all that the patient can afford. In Peru and central Thailand, private retail pharmacies, where dispensing could not be guided by the antibiotic-resistance profiles of the causative agents, were found to be the most common source of antibiotics for the treatment of sexually transmitted diseases.^25,26^

In Thailand, we identified about 24 000 distributors, retailers and wholesalers who were fully licensed for pharmaceutical sales in 2017. At the time of our study, the records of the Thai Food and Drug Administration did not differentiate between such licensed distributors, retailers and wholesalers. In consequence, there was no easy way to monitor or control the sale of large quantities of antibiotics to individual patients or farmers. We found that, if they could afford it, Thai farmers could easily buy very large amounts of finished products and active pharmaceutical ingredients from drug retailers or wholesalers.

### Regulatory environment

The focus of drug regulation in low- and middle-income countries, e.g. Ethiopia, Thailand, the United Republic of Tanzania and Zimbabwe, is on drug quality and licensing rather than availability and distribution channels.^10–12^

In Thailand, the 1987 Drug Act did attempt to regulate the availability of some antibiotics, by dividing antibiotics into a large group of “dangerous drugs not requiring prescriptions” and a much smaller group of “special-control drugs requiring prescriptions”.^30^ This categorization meant that most antibiotics could be dispensed, by licensed pharmacists in retail pharmacies, without a prescription. Furthermore, the Act made no attempt to regulate the quantity of antibiotics that could be distributed to any individual or to control the excessive use of antibiotics in livestock. Later, the 2015 Animal Feed Quality Control Act prohibited direct use of active pharmaceutical ingredient in the animal feeds. However, our interviews indicated that, many Thai farmers were, illegally, adding active pharmaceutical ingredients to animal feeds, probably as a cost-saving measure.

Following a series of public consultations, the Thai Food and Drug Administration is working on a reclassification of antibiotics in which a larger proportion of the drugs will be categorized as special-control/prescription-only, in line with the recommendations made by the World Health Organization in its 20th Model List of Essential Medicines.^27^

Compared with access to antibiotics, access to active pharmaceutical ingredients appears to be less well regulated, leading to inappropriate use by farmers. In Thailand, all drugs have to be registered with the Food and Drug Administration before production or importation. There is, however, no corresponding requirement for the registration of active pharmaceutical ingredients. Drug distributors and retailers can only sell active pharmaceutical ingredients legally to manufacturers. However, a lack of monitoring and tracking of active pharmaceutical ingredients and inadequate inspections at the drug distributors and retailers mean that this legal restriction is generally ignored.

One limitation of our study is that the data maintained by the Thai Food and Drug Administration do not allow any estimation of the national consumption of each major class of antibiotics in terms of, for example, the defined daily dose per 1000 inhabitants per day. The Thai Working Group on the Surveillance of Antimicrobial Consumption is working on the development of a sustainable system to monitor annual antimicrobial consumption.^28^

In conclusion, this study appears to be the first published study in Thailand to investigate antibiotic distribution, for human and animal health. The thousands of drug distributors, drug wholesalers, retail pharmacies and animal feed stores that have arisen in the country, as a result of market forces, and the small number of antibiotics that are classified as special-control/prescription-only make most antibiotics easily and widely available in both the human and animal health sectors. Such wide availability probably leads to frequent inappropriate use. A general lack of enforcement of the legislation covering the distribution of active pharmaceutical ingredients facilitates the direct use of such ingredients on farms.

The unnecessary and inappropriate use of antibiotics will probably lead to an increase in the problem posed by antimicrobial resistance in Thailand. A system for recording antibiotic dispensing at retail pharmacies should be established^29^ and then carefully audited by pharmacists. The continued professional education of retail pharmacists should be promoted, as a means of reducing the inappropriate use of antibiotics, and other drugs. The sales of large quantities of antibiotics to individuals need to be restricted by differentiating wholesalers from retailers in the licensing system. This includes prohibiting wholesalers from selling large quantities of antibiotics to farmers, or others who are not licensed retail outlets, and carefully restricting the sale by retailers of large quantities of such drugs to individuals. The ongoing policy to reclassify more antibiotics as special-control/prescription-only drugs in Thailand should be rapidly implemented. A national system for tracking active pharmaceutical ingredients should be established immediately, to prevent the direct use of such ingredients on farms. 
